# Re-evaluating large for gestational age: differential effects on perinatal outcomes in term and premature births

**DOI:** 10.3389/fmed.2024.1498712

**Published:** 2025-01-15

**Authors:** Chun-Heng Kuo, Yi-Ling Wu, Chi-Nien Chen, Yu-Ru Lo, I-Weng Yen, Kang-Chih Fan, Yi-Yun Tai, Ming-Wei Lin, Chih-Cheng Hsu, Hung-Yuan Li

**Affiliations:** ^1^Graduate Institute of Clinical Medicine, College of Medicine, National Taiwan University, Taipei, Taiwan; ^2^School of Medicine, College of Medicine, Fu Jen Catholic University, New Taipei City, Taiwan; ^3^Department of Internal Medicine, Fu Jen Catholic University Hospital, Fu Jen Catholic University, New Taipei City, Taiwan; ^4^Institute of Population Health Sciences, National Health Research Institutes, Miaoli, Taiwan; ^5^Department of Pediatrics, National Taiwan University Hospital, Hsin-Chu Branch, Hsinchu, Taiwan; ^6^Department of Pediatrics, National Taiwan University College of Medicine, Taipei, Taiwan; ^7^Division of Endocrinology and Metabolism, Department of Internal Medicine, National Taiwan University Hospital, Hsin-Chu Branch, Hsinchu, Taiwan; ^8^Department of Medical Genetics, National Taiwan University Hospital, Taipei, Taiwan; ^9^Department of Obstetrics and Gynecology, National Taiwan University Hospital, Hsin-Chu Branch, Hsinchu, Taiwan; ^10^Department of Family Medicine, Min-Sheng General Hospital, Taoyuan, Taiwan; ^11^Department of Health Services Administration, China Medical University, Taichung, Taiwan; ^12^National Center for Geriatrics and Welfare Research, National Health Research Institutes, Yunlin, Taiwan; ^13^Division of Endocrinology and Metabolism, Department of Internal Medicine, National Taiwan University Hospital, Taipei, Taiwan

**Keywords:** large for gestational age, perinatal outcomes, preterm, prematurity, fetal death, neonatal death

## Abstract

**Objective:**

Pregnancies with large-for-gestational-age (LGA) fetuses are associated with increased risks of various adverse perinatal outcomes. While existing research primarily focuses on term neonates, less is known about preterm neonates. This study aims to explore the risks of adverse maternal and neonatal perinatal outcomes associated with LGA in term neonates and neonates with different degrees of prematurity, compared to appropriate-for-gestational-age (AGA) neonates.

**Methods:**

Using the Birth Reporting Databases (2007–2018) linked to Taiwan's National Health Insurance Research Database, we conducted a retrospective nationwide cohort study of singleton neonates delivered between 24 and 42 weeks of gestation. Based on gestational age at delivery, the enrolled neonates were classified into term (37–42 weeks of gestation), late preterm (34–36 weeks of gestation), moderate preterm (32–33 weeks of gestation), very preterm (28–31 weeks of gestation), and extremely preterm (24–27 weeks of gestation). LGA was defined by the 2013 World Health Organization (WHO) growth standard and the Taiwan growth standard. Perinatal outcomes were compared between LGA and AGA neonates across different gestational age groups.

**Results:**

Among the 1,602,638 neonates, 44,359 were classified as LGA by the 2013 WHO growth standard. Compared to AGA neonates, LGA neonates in term and late preterm groups exhibited higher risks of primary cesarean section, prolonged labor, neonatal hypoglycemia, birth trauma, hypoxic ischemic encephalopathy, jaundice needing phototherapy, respiratory distress, neonatal intensive care unit (NICU) admission, newborn sepsis, and fetal death. However, most of these risks were not increased in moderate, very, and extremely preterm groups. Conversely, being LGA was associated with lower risks of primary cesarean section (very preterm group), jaundice needing phototherapy (moderate and very preterm groups), respiratory distress (moderate and very preterm groups), NICU admission (moderate and very preterm groups), newborn sepsis (very preterm group), retinopathy of prematurity (late, moderate, and very preterm groups), and bronchopulmonary dysplasia (very preterm group). These findings remained consistent when the Taiwan growth standard was applied.

**Conclusion:**

Being LGA is associated with increased risks of perinatal complications in term and late preterm neonates, but not in earlier preterm groups. These findings underscore the importance of tailoring management strategies for LGA neonates to consider different degrees of prematurity.

## 1 Introduction

Pregnancies with large-for-gestational-age (LGA) fetuses are associated with many short-term (1–3) and long-term (4–6) adverse complications. During the perinatal period, laboring a LGA fetus increases the risks of cesarean section, prolonged labor, perineal lacerations, postpartum hemorrhage, birth trauma, neonatal hypoglycemia, neonatal jaundice, respiratory distress syndrome (RDS), neonatal intensive care unit (NICU) admission, and even fetal and neonatal death (1–3). In childhood, LGA neonates have higher chances of developing metabolic disorders, including obesity, hypertension, insulin resistance, and type 2 diabetes mellitus (4–6). However, most of these findings have been derived from studies focused on term neonates (1–6).

For preterm neonates, it might be expected that a higher birth weight would offer some advantages. Indeed, several studies have found that being LGA in preterm neonates does not increase the risk of certain prematurity-related morbidities, such as necrotizing enterocolitis (NEC), newborn sepsis, intraventricular hemorrhage (IVH), and retinopathy of prematurity (ROP) (7–10). However, these studies primarily examined outcomes related to prematurity, without evaluating the maternal and neonatal complications specifically associated with LGA in preterm neonates. Given that LGA-related morbidities are often attributed to large fetal size, we hypothesize that the risks associated with LGA differ between term and preterm neonates. If this hypothesis holds true, the current strategies in managing for LGA neonates may need to be refined to better address the unique challenges associated with prematurity. Thus, the aim of the present study is to investigate the risks of adverse maternal and neonatal outcomes associated with LGA in neonates born at term and at different degrees of prematurity, compared to those born appropriate-gestational-age (AGA).

## 2 Materials and methods

### 2.1 Data source

This is a retrospective nationwide cohort study. The Birth Reporting Databases (BRD) in 2007–2018 connected to National Health Insurance Research Database (NHIRD) from the Health and Welfare Data Science Center (HWDC) were used in the present study. The birth weight and gestational age of neonates are contained in the BRD. To investigate neonatal outcomes, we used the Maternal and Child Health Database which was generated from BRD, Birth Registration Database, National Register of Death, and the NHIRD by HWDC. Data of pregnancy outcomes were obtained from NHIRD. The study was approved by the Institutional Review Board (IRB) of National Health Research Institutes (EC1110505-E). Because personal information in the NHIRD was encrypted before release, the IRB granted a waiver of informed consent.

### 2.2 Study population

Every neonate delivered in Taiwan was reported to the BRD by health organizations. Singletons ≥24 and ≤ 42 weeks of gestational age at delivery in the BRD in 2007–2018 were selected as the study population in this study. According to different gestational age, neonatal sex, and birth weight, these singletons were classified into three groups: LGA, AGA, and small for gestational age (SGA). LGA was defined as a birth weight above the 90th percentile for a given gestational age and neonatal sex, based on the 2013 WHO growth standards (11) or the Taiwan growth standards (12). SGA was defined as a birth weight below the 10th percentile for a given gestational age and neonatal sex, while AGA was defined as a birth weight between the 10th and 90th percentiles for a given gestational age and neonatal sex. Only singletons classified as LGA or AGA were included. We excluded maternal age < 18 or older than 55 years, and the neonates with unknown sex. We also excluded the pairs with mismatched information in neonate's birth date and corresponding mother's delivery date. Additionally, neonates whose birth conditions could not be clearly identified, including pregnant women having twice deliveries in a year, neonates without identification, and those who could not be followed for at least 1 year were also excluded. Based on gestational age at delivery, the enrolled neonates were classified into five groups (13, 14), which were term (≥37 and ≤ 42 weeks), late preterm (≥34 and <37 weeks), moderate preterm (≥32 and <34 weeks), very preterm (≥28 and <32 weeks), and extremely preterm (≥24 and <28 weeks). Maternal baseline information, including age, neonatal sex, gestational age, gestational diabetes mellitus (GDM), preexisting diabetes mellitus, newly diagnosed diabetes mellitus in pregnancy, gestational hypertension (GH), preeclampsia, hypertension, polycystic ovary syndrome (PCOS), living area, family income, and babies' birth weight, were also collected.

### 2.3 Outcome measurements

Common adverse pregnancy outcomes identified during the hospitalization for delivery including primary cesarean section and prolonged or obstructed labor were assessed. Neonatal outcomes, including neonatal hypoglycemia, birth trauma, hypoxic ischemic encephalopathy (HIE), jaundice, and respiratory distress were identified within 1 month of delivery. Admission to neonatal intensive care unit (NICU), fetal death, and neonatal death were also collected. Fetal death is defined as spontaneous intrauterine death of a fetus at any time during pregnancy and prior to delivery (15). Neonatal death is defined as death within 28 days after birth (16). Common adverse outcomes in prematurity were identified within 4 months of delivery, including newborn sepsis, ROP, IVH, NEC, and bronchopulmonary dysplasia (BPD). All outcome measurements were assessed using International Classification of Diseases (ICD) codes.

### 2.4 Statistical analysis

Categorical variables were presented as percentages, and continuous variables were summarized by means and standard deviations. The baseline characteristics between LGA and AGA groups were compared using Student's *t*-test for continuous variables and the chi-squared test for categorical variables. Incidences of pregnancy outcomes were calculated in both LGA and AGA groups based on different gestational age at delivery. Outcomes were also compared between the LGA and AGA groups and presented in odds ratios (ORs) and adjusted ORs with 95% confidence intervals (95% CIs). The multivariable models were adjusted for maternal age, gestational age, neonatal sex, diabetes in pregnancy (DIP, defined as GDM, preexisting diabetes mellitus, or newly diagnosed diabetes mellitus), hypertensive disorders of pregnancy (defined as GH, preeclampsia, or chronic hypertension), PCOS, living area, and monthly family income. All statistical analyses were conducted with SAS software version 9.4 (SAS Institute Inc., Cary, NC). A two-sided *P*-value < 0.05 was considered statistically significant.

## 3 Results

### 3.1 Study patients

**Figure 1:**
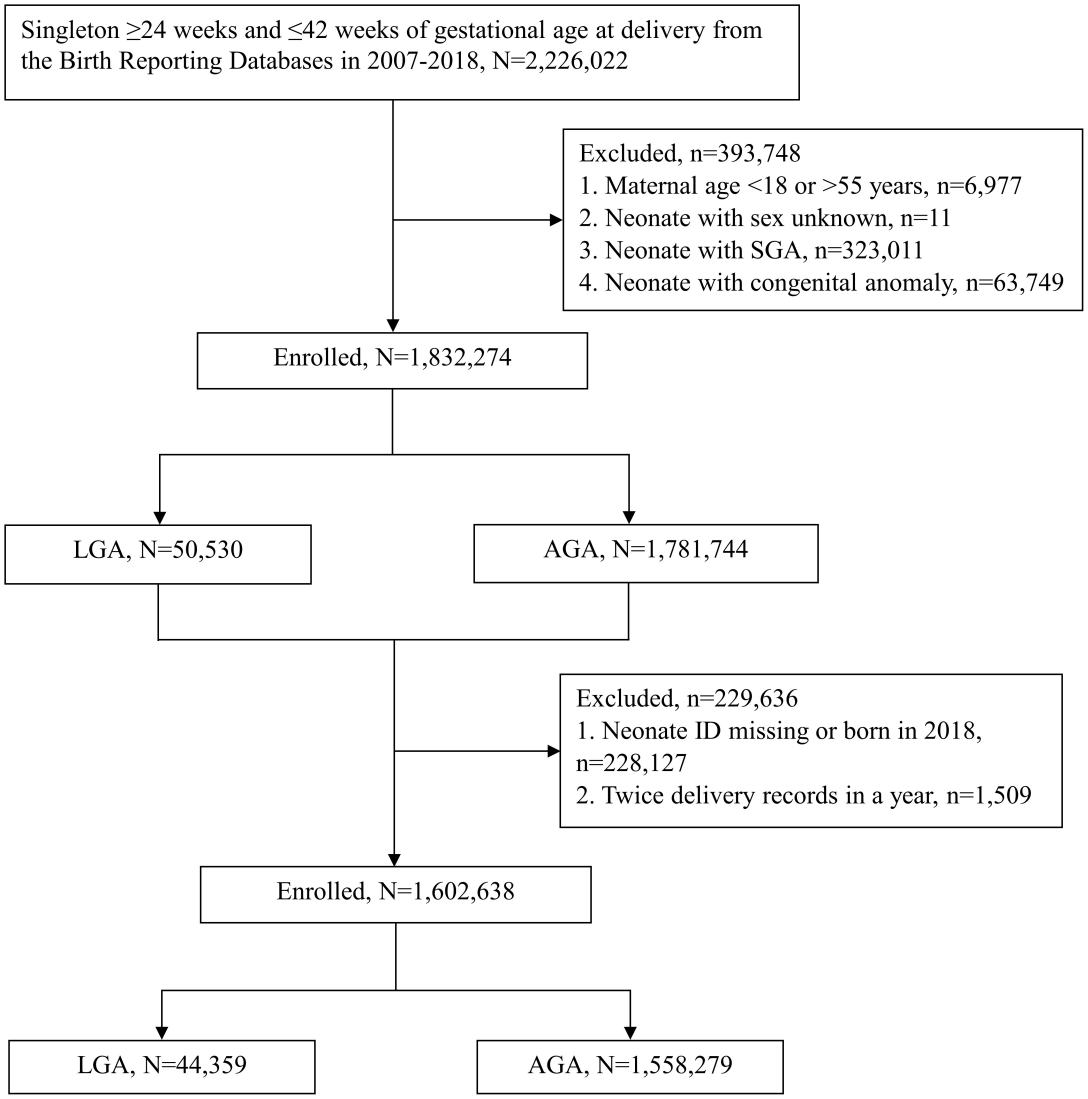
Flowchart depicting enrollment of the participants in the study. Large for gestational age (LGA) and appropriate for gestational age (AGA) are defined by the WHO growth standard. SGA, small for gestational age; WHO, World Health Organization.

From 1 January, 2007 to 31 December, 2018, a total of 2,226,022 singleton neonates with gestational age at delivery ≥24 and ≤ 42 weeks were found in the BRD. Of these neonates, we excluded 6,977 whose mothers' age were <18 or older than 55 years, 11 whose sex were not recorded, 323,011 who were classified as SGA, and 63,749 with congenital anomaly. Among the remaining 1,832,274 neonates, we deleted 228,127 neonates whose ID were missing or who were born in 2018, and 1,509 neonates whose characteristics could not be clearly identified due to their mother having twice delivery in a year. Finally, 1,602,638 neonates were eligible for analysis, including 44,359 in the LGA group and 1,558,279 in the AGA group (Figure 1).

### 3.2 Baseline characteristics

The baseline characteristics including maternal age, gestational age at delivery, birth weight of newborn, comorbidities of the pregnant women, living area, and family income are summarized in Table 1. The mean maternal age between the LGA and AGA groups were 32.25 vs. 31.01 years in the whole study population. Subjects were classified into five groups according to gestational age at birth: group 1 (term, ≥37 and ≤ 42 weeks), group 2 (late preterm, ≥34 and <37 weeks), group 3 (moderate preterm, ≥32 and <34 weeks), group 4 (very preterm, ≥28 and <32 weeks), and group 5 (extremely preterm, ≥24 and <28 weeks). In groups 1–2, the mean maternal age in the LGA subgroup was significantly older when compared to the AGA subgroup. As shown in Table 1, the proportions of GDM between the LGA and AGA groups were 20.49 vs. 12.50% in the whole study population. Groups 1–4 had significantly higher proportions of GDM, preexisting diabetes mellitus, and newly diagnosed diabetes mellitus in the LGA subgroup than in the AGA subgroup. Compared to the AGA subgroup, groups 1–2 had higher proportions of GH, preeclampsia, HTN and PCOS in the LGA subgroup. However, lower proportions of GH, preeclampsia, and HTN were seen in the LGA subgroup in group 4, and preeclampsia also in group 5, compared to the AGA subgroup. Additionally, a significantly different distribution in the living area and monthly family income were observed in the LGA subgroup compared to the AGA subgroup in each group.

**Table 1:** Baseline characteristics of pregnant women with LGA neonates and AGA neonates, divided into 5 groups according to gestational age (GA) at delivery.^*^

	**Group 1**			**Group 2**			**Group 3**			**Group 4**			**Group 5**		
	**GA 37–42 weeks**			**GA 34–36 weeks**			**GA 32–33 weeks**			**GA 28–31 weeks**			**GA 24–27 weeks**		
	**LGA**	**AGA**	***p*** **-value**	**LGA**	**AGA**	***p*** **-value**	**LGA**	**AGA**	***p*** **-value**	**LGA**	**AGA**	***p*** **-value**	**LGA**	**AGA**	***p*** **-value**
*N* (%)	38,305 (2.57)	1,449,369 (97.43)		4,927 (5.01)	93,323 (94.99)		502 (5.77)	8,197 (94.23)		490 (8.14)	5,528 (91.86)		135 (6.76)	1,862 (93.24)	
Maternal age (years)	32.27 ± 4.67	31.03 ± 4.70	**< 0.0001**	32.66 ± 4.95	31.29 ± 5.07	**< 0.0001**	31.67 ± 5.56	31.69 ± 5.22	0.9309	31.90 ± 5.67	32.08 ± 5.20	0.4912	32.16 ± 5.63	32.09 ± 5.19	0.8831
Neonatal sex (male, %)	49.63	51.58	**< 0.0001**	57.60	56.69	0.2091	53.59	57.87	0.0590	60.41	56.58	0.1014	48.15	56.93	**0.0470**
GA (weeks)	38.29 ± 1.02	38.57 ± 1.04	**< 0.0001**	35.56 ± 0.67	35.57 ± 0.67	0.6389	32.57 ± 0.50	32.61 ± 0.49	**0.0313**	29.72 ± 1.08	29.87 ± 1.09	**0.0036**	25.87 ± 1.13	25.75 ± 1.11	0.2271
BW (g)	4,044 ± 261	3,194 ± 298	**< 0.0001**	3,546 ± 346	2,643 ± 307	**< 0.0001**	2,748 ± 314	1,972 ± 256	**< 0.0001**	1,967 ± 357	1,434 ± 264	**< 0.0001**	1,207 ± 281	858 ± 159	**< 0.0001**
GDM (%)	20.04	12.45	**< 0.0001**	24.98	13.40	**< 0.0001**	19.72	13.68	**0.0007**	15.51	11.25	**0.0053**	5.93	3.97	0.3462
Preexisting diabetes mellitus (%)	2.97	0.48	**< 0.0001**	9.07	1.00	**< 0.0001**	8.96	1.67	**< 0.0001**	6.12	2.13	**< 0.0001**	2.96	1.34	0.2023
Newly diagnosed diabetes mellitus in pregnancy (%)	0.82	0.16	**< 0.0001**	2.39	0.32	**< 0.0001**	2.79	0.45	**< 0.0001**	1.63	0.43	**0.0008**	2.22	0.38	**0.0086**
GH (%)	4.14	1.86	**< 0.0001**	10.07	4.57	**< 0.0001**	8.96	7.98	0.4304	*5.71*	*10.38*	***0.0010***	4.44	5.85	0.4973
Preeclampsia (%)	3.31	1.43	**< 0.0001**	8.18	4.51	**< 0.0001**	7.57	10.09	0.0671	*4.08*	*13.12*	*** < 0.0001*** ^‡^	*1.48*	*6.98*	***0.0130*** ^‡^
HTN (%)	2.37	1.18	**< 0.0001**	6.64	3.43	**< 0.0001**	9.16	7.42	0.1498	*3.67*	*10.60*	*** < 0.0001*** ^‡^	3.70	6.93	0.1482
PCOS (%)	1.39	1.10	**< 0.0001**	1.75	1.40	**0.0437**	1.79	1.52	0.6362	1.02	1.83	0.1933	0.00	1.61	0.1373
**Living area**^†^ **(%)**															
Urban	74.25	75.	**< 0.0001**	70.35	74.	**< 0.0001**	76.29	82.	**0.0036**	79.59	87.	**< 0.0001**	80.74	87.	**0.0476**
Suburban	22.49	22.05		26.39	22.57		19.72	14.53		16.73	9.70		14.07	8.11	
Rural	3.13	2.36		3.15	2.48		3.98	2.76		3.67	2.84		5.19	4.40	
**Monthly family income (NTD) (%)**															
≤ 20,100	19.37	19	**< 0.0001**	24.09	22	**< 0.0001**	29.88	23	**< 0.0001**	27.76	22	**0.0026**	31.85	21	**0.0195**
20,101–22,800	24.01	23.64		26.06	24.16		21.51	23.11		20.61	23.77		22.22	23.52	
22,801–42,000	28.55	31.51		24.78	29.85		24.90	29.14		28.98	29.31		24.44	30.13	
>42,000	19.09	20.53		13.88	18.54		14.54	18.93		14.69	19.01		12.59	18.69	

Definitions of LGA and AGA are according to the WHO growth standard. Data were presented as mean ± standard deviation or percentage.

AGA, appropriate for gestational age; BW, birth weight; GA, gestational age; GDM, gestational diabetes mellitus; GH, gestational hypertension; HTN, hypertension; LGA, large for gestational age; NTD, New Taiwan Dollar; PCOS, polycystic ovary syndrome; WHO, World Health Organization.

^*^Group 1: term (gestational age ≥37 and ≤ 42 weeks); Group 2: late preterm (gestational age ≥34 and < 37 weeks); Group 3: moderate preterm (gestational age ≥32 and < 34 weeks); Group 4: very preterm (gestational age ≥28 and < 32 weeks); Group 5: extremely preterm (≥24 and < 28 weeks).

^†^Urban (metropolis), suburban (satellite cities), exurban or rural areas.

^‡^Italic values denote the variable is significantly lower in the LGA subgroup than in the AGA subgroup. Bold values indicate p-values < 0.05.

### 3.3 Analysis of common adverse pregnancy and neonatal outcomes

The incidence of common adverse pregnancy and neonatal outcomes in both LGA and AGA subgroups among 5 different gestational-age groups were presented in Supplementary Table 1. When compared to the AGA subgroup (Table 2), the LGA subgroup was associated with higher risks of primary cesarean section in group 1 (OR 2.18, 95% CI 2.13–2.22) and group 2 (OR 1.30, 95% CI 1.22–1.38), but the risk was lower in group 4 (OR 0.62, 95% CI 0.52–0.75). The LGA subgroup had higher risks in prolonged or obstructed labor in group 1 (OR 1.54, 95% CI 1.49–1.59) and group 2 (OR 1.74, 95% CI 1.53–1.99), compared with the AGA subgroup. In groups 1–2, the LGA subgroup had higher risks than the AGA subgroup for neonatal hypoglycemia (OR 9.00, 95% CI 8.19–9.89; OR 2.05, 95% CI 1.75–2.41), birth trauma (OR 4.62, 95% CI 4.20–5.09; OR 6.75, 95% CI 4.83–9.42), and HIE (OR 1.64, 95% CI 1.28–2.09; OR 1.67, 95% CI 1.11–2.52). The LGA subgroup also had higher risks for jaundice needing phototherapy/exchange transfusion, respiratory distress, and NICU admission in group 1 (OR 1.06, 95% CI 1.04–1.08; OR 2.27, 95% CI 2.00–2.57; OR 1.85, 95% CI 1.75–1.97, respectively) and group 2 (OR 1.13, 95% CI 1.06–1.19; OR 1.15, 95% CI 1.01–1.30; OR 1.40, 95% CI 1.29–1.53, respectively), but lower risks in group 3 (OR 0.61, 95% CI 0.51–0.74; OR 0.66, 95% CI 0.54–0.81; OR 0.69, 95% CI 0.58–0.83, respectively) and group 4 (OR 0.51, 95% CI 0.42–0.62; OR 0.65, 95% CI 0.54–0.78; OR 0.45, 95% CI 0.36–0.56, respectively). The risk of fetal death was higher in the LGA subgroup than in the AGA subgroup among the five groups, and the risk of fetal death associated with LGA was the highest in group 1. Groups 1–4 had significant higher risks of neonatal death in the LGA subgroup than in the AGA subgroup.

**Table 2:** Crude odds ratios (95% confidence intervals) for pregnancy outcomes of pregnant women with LGA neonates, compared to pregnant women with AGA neonates, divided into 5 groups according to gestational age (GA) at delivery.^*^

	**Group 1**	**Group 2**	**Group 3**	**Group 4**	**Group 5**
	**GA 37–42 weeks**	**GA 34–36 weeks**	**GA 32–33 weeks**	**GA 28–31 weeks**	**GA 24–27 weeks**
**Common adverse pregnancy outcomes**					
Primary cesarean section	**2.18 (2.13–2.22)** ^**§**^	**1.30 (1.22–1.38)** ^**§**^	0.97 (0.81–1.17)	***0.62 (0.52–0.75)** ^**§¶**^*	0.73 (0.51–1.05)
Prolonged or obstructed labor	**1.54 (1.49–1.59)** ^**§**^	**1.74 (1.53–1.99)** ^**§**^	1.79 (0.77–4.18)	1.41 (0.32–6.16)	NA
**Common adverse neonatal outcomes**					
Neonatal hypoglycemia	**9.00 (8.19–9.89)** ^**§**^	**2.05 (1.75–2.41)** ^**§**^	1.29 (0.87–1.94)	1.58 (0.96–2.62)	1.20 (0.28–5.16)
Birth trauma (shoulder dystocia, brachial plexus injury, clavicular fracture)	**4.62 (4.20–5.09)** ^**§**^	**6.75 (4.83–9.42)** ^**§**^	2.34 (0.29–19.02)	NA	NA
Hypoxic ischemic encephalopathy	**1.64 (1.28–2.09)** ^**§**^	**1.67 (1.11–2.52)** ^**†**^	1.55 (0.71–3.39)	0.53 (0.17–1.68)	1.09 (0.33–3.58)
Jaundice needing phototherapy or exchange transfusion	**1.06 (1.04–1.08)** ^**§**^	**1.13 (1.06–1.19)** ^**§**^	***0.61 (0.51–0.74)** ^**§¶**^*	***0.51 (0.42–0.62)** ^**§¶**^*	0.96 (0.65–1.40)
Respiratory distress	**2.27 (2.00–2.57)** ^**§**^	**1.15 (1.01–1.30)** ^**†**^	***0.66 (0.54–0.81)** ^**§¶**^*	***0.65 (0.54–0.78)** ^**§¶**^*	0.73 (0.51–1.05)
NICU admission	**1.85 (1.75–1.97)** ^**§**^	**1.40 (1.29–1.53)** ^**§**^	***0.69 (0.58–0.83)** ^**§¶**^*	***0.45 (0.36–0.56)** ^**§¶**^*	0.78 (0.46–1.32)
Fetal death^||^	**3.86 (2.86–5.19)** ^**§**^	**2.48 (1.79–3.44)** ^**§**^	**2.04 (1.29–3.23)** ^**‡**^	**1.58 (1.13–2.23)** ^**‡**^	**2.14 (1.55–2.96)** ^**§**^
Neonatal death ( ≤ 28 days)	**1.69 (1.11–2.56)** ^**†**^	**1.74 (1.02–2.95)** ^**†**^	**3.22 (1.87–5.54)** ^**§**^	**1.84 (1.27–2.68)** ^**‡**^	1.10 (0.76–1.59)
**Common adverse outcomes in prematurity**					
Newborn sepsis	**1.22 (1.16–1.29)** ^**§**^	**1.15 (1.04–1.28)** ^**‡**^	0.93 (0.74–1.17)	0.81 (0.66–1.01)	1.12 (0.78–1.60)
Retinopathy of prematurity	NA	***0.29 (0.14–0.58)** ^**§¶**^*	***0.49 (0.34–0.71)** ^**§¶**^*	***0.62 (0.49–0.79)** ^**§¶**^*	0.91 (0.60–1.38)
Intraventricular hemorrhage	1.44 (0.89–2.34)	1.15 (0.70–1.87)	0.54 (0.28–1.06)	0.89 (0.61–1.31)	1.26 (0.73–2.17)
Necrotizing enterocolitis	1.07 (0.40–2.90)	0.42 (0.10–1.69)	0.39 (0.10–1.60)	0.96 (0.56–1.63)	0.38 (0.12–1.22)
Bronchopulmonary dysplasia	1.40 (0.34–5.75)	0.82 (0.11–6.10)	1.49 (0.46–4.87)	***0.55 (0.37–0.81)** ^**‡¶**^*	0.66 (0.42–1.05)

Definitions of LGA and AGA are according to the WHO growth standard.

AGA, appropriate for gestational age; LGA, large for gestational age; NA, not applicable due to rare events of the outcome in this group; NICU, neonatal intensive care unit; PDA, patent ductus arteriosus; WHO, World Health Organization.

^*^Group 1: term (gestational age ≥37 and ≤ 42 weeks); Group 2: late preterm (gestational age ≥34 and < 37 weeks); Group 3: moderate preterm (gestational age ≥32 and < 34 weeks); Group 4: very preterm (gestational age ≥28 and < 32 weeks); Group 5: extremely preterm (≥24 and < 28 weeks).

^†^*p*-value < 0.05.

^‡^*p*-value < 0.01.

^§^*p*-value < 0.001.

^||^Subjects with missing neonatal ID, subjects whose neonates were born in 2018, and subjects with twice delivery records in a year were included for analysis.

^¶^Italic values denote the odds ratio is below 1. Bold values indicate odds ratios with p-value < 0.05.

In Table 3, we analyzed the adjusted ORs associated with LGA, and most adjusted ORs showed the consistent results to the unadjusted ORs, except that the LGA subgroup in group 2 was not significantly associated with risks of HIE (adjusted OR 1.45, 95% CI 0.95–2.21), respiratory distress (adjusted OR 1.00, 95% CI 0.88–1.14), and neonatal death (adjusted OR 1.58, 95% CI 0.92–2.72). We further performed subgroup analyses in subjects with or without DIP (Supplementary Tables 6–9). The overall risk patterns were similar between subjects with and without DIP, except that the protective roles of LGA on jaundice, respiratory distress and NICU admission in groups 3 and 4 were not observed in pregnant women with DIP.

**Table 3:** Adjusted odds ratios (95% confidence intervals) for pregnancy outcomes of pregnant women with LGA neonates, compared to pregnant women with AGA neonates, divided into 5 groups according to gestational age (GA) at delivery.^*^

	**Group 1**	**Group 2**	**Group 3**	**Group 4**	**Group 5**
	**GA 37–42 weeks**	**GA 34–36 weeks**	**GA 32–33 weeks**	**GA 28–31 weeks**	**GA 24–27 weeks**
**Common adverse pregnancy outcomes**			
Primary cesarean section	**2.13 (2.09–2.18)** ^**§**^	**1.15 (1.08–1.23)** ^**§**^	0.98 (0.80–1.18)	***0.69 (0.57–0.84)** ^**§¶**^*	0.76 (0.53–1.11)
Prolonged labor or obstructed labor	**1.64 (1.61–1.68)** ^**§**^	**1.75 (1.58–1.94)** ^**§**^	**2.77 (1.42–5.41)** ^**‡**^	1.38 (0.31–6.12)	NA
**Common adverse neonatal outcomes**			
Neonatal hypoglycemia	**7.26 (6.59–7.99)** ^**§**^	**1.73 (1.47–2.05)** ^**§**^	1.32 (0.88–1.99)	1.57 (0.94–2.61)	1.22 (0.28–5.36)
Birth trauma (shoulder dystocia, brachial plexus injury, clavicular fracture)	**4.31 (3.91–4.76)** ^**§**^	**6.31 (4.44–8.98)** ^**§**^	1.83 (0.22–15.40)	NA	NA
Hypoxic ischemic encephalopathy	**1.56 (1.22–1.99)** ^**§**^	1.45 (0.95–2.21)	1.78 (0.81–3.91)	0.52 (0.16–1.68)	1.01 (0.30–3.41)
Jaundice needing phototherapy or exchange transfusion	**1.03 (1.01–1.06)** ^**‡**^	**1.07 (1.00–1.13)** ^**†**^	***0.60 (0.50–0.73)** ^**§¶**^*	***0.52 (0.42–0.63)** ^**§¶**^*	1.03 (0.70–1.51)
Respiratory distress	**2.06 (1.81–2.34)** ^**§**^	1.00 (0.88–1.14)	***0.65 (0.53–0.80)** ^**§¶**^*	***0.65 (0.54–0.78)** ^**§¶**^*	0.77 (0.53–1.11)
NICU admission	**1.74 (1.64–1.85)** ^**§**^	**1.26 (1.16–1.37)** ^**§**^	***0.68 (0.57–0.82)** ^**§¶**^*	***0.47 (0.37–0.59)** ^**§¶**^*	0.85 (0.49–1.45)
Fetal death^||^	**3.16 (2.34–4.27)** ^**§**^	**2.29 (1.63–3.20)** ^**§**^	**1.95 (1.93–1.97)** ^§^	**1.52 (1.07–2.15)** ^**†**^	**1.92 (1.37–2.71)** ^**§**^
Neonatal death ( ≤ 28 days)	**1.58 (1.04–2.40)** ^**†**^	1.58 (0.92–2.72)	**3.43 (1.98–5.97)** ^§^	**1.86 (1.27–2.73)** ^**‡**^	1.04 (0.71–1.53)
**Common adverse outcomes in prematurity**			
Newborn sepsis	**1.19 (1.13–1.26)** ^**§**^	1.08 (0.98–1.20)	0.89 (0.71–1.12)	***0.75 (0.61–0.93)** ^**‡¶**^*	1.10 (0.76–1.58)
Retinopathy of prematurity	NA	***0.30 (0.15–0.59)** ^**§¶**^*	***0.50 (0.35–0.73)** ^**§¶**^*	***0.63 (0.50–0.80)** ^**§¶**^*	0.92 (0.60–1.40)
Intraventricular hemorrhage	1.34 (0.82–2.17)	1.00 (0.61–1.64)	0.56 (0.29–1.11)	0.86 (0.59–1.27)	1.29 (0.74–2.24)
Necrotizing enterocolitis	1.07 (0.40–2.91)	0.41 (0.10–1.66)	0.35 (0.08–1.41)	0.89 (0.52–1.53)	0.36 (0.11–1.15)
Bronchopulmonary dysplasia	1.36 (0.33–5.62)	0.99 (0.13–7.41)	1.68 (0.51–5.57)	***0.59 (0.40–0.88)** ^**‡¶**^*	0.74 (0.46–1.19)

Definitions of LGA and AGA are according to the WHO growth standard.

AGA, appropriate for gestational age; LGA, large for gestational age; NA, not applicable due to rare events of the outcome in this group; NICU, neonatal intensive care unit; WHO, World Health Organization.

Models were adjusted for maternal age, gestational age, neonatal sex, diabetes in pregnancy (defined as gestational diabetes mellitus, preexisting diabetes mellitus, or newly diagnosed diabetes mellitus), hypertensive disorders of pregnancy (defined as gestational hypertension, preeclampsia, or chronic hypertension), polycystic ovary syndrome, living area, and monthly family income.

^*^Group 1: term (gestational age ≥37 and ≤ 42 weeks); Group 2: late preterm (gestational age ≥34 and < 37 weeks); Group 3: moderate preterm (gestational age ≥32 and < 34 weeks); Group 4: very preterm (gestational age ≥28 and < 32 weeks); Group 5: extremely preterm (≥24 and < 28 weeks).

^†^*p*-value < 0.05.

^‡^*p*-value < 0.01.

^§^*p*-value < 0.001.

^||^Subjects with missing neonatal ID, subjects whose neonates were born in 2018, and subjects with twice delivery records in a year were included for analysis.

^¶^Italic values denote the odds ratio is below 1. Bold values indicate odds ratios with p-value < 0.05.

### 3.4 Analysis of common adverse outcomes in prematurity

In Table 2, the risk of newborn sepsis in the LGA subgroup comparing to the AGA subgroup was higher in group 1 (OR 1.22, 95% CI 1.16–1.29) and group 2 (OR 1.15, 95% CI 1.04–1.28). For ROP, excluding not applicable data, the LGA subgroup was associated with lower risks in group 2 (OR 0.29, 95% CI 0.14–0.58), group 3 (OR 0.49, 95% CI 0.34–0.71), and group 4 (OR 0.62, 95% CI 0.49–0.79) while comparing to the AGA subgroups. Besides, the LGA subgroup had lower risks of BPD in group 4 (OR 0.55, 95%CI 0.37–0.81) when compared to the AGA subgroup. The adjusted ORs for these adverse outcomes in prematurity showed similar results to the unadjusted ORs (Table 3), except that the risk of newborn sepsis in the LGA subgroup was not significantly increased in group 2 (adjusted OR 1.08, 95% CI 0.98–1.20) and was significantly decreased in group 4 (adjusted OR 0.75, 95% CI 0.61–0.93) when compared with the AGA subgroup.

### 3.5 Analyses using LGA and AGA definitions based on Taiwan growth standard

When the definition of LGA was based on Taiwan growth standard, the results were similar with those based on the 2013 WHO growth standard. The flow chart is shown in Supplementary Figure 1, and the baseline characteristics are summarized in Supplementary Table 2. When compared to the AGA subgroup, the LGA subgroup had significantly older maternal age and higher rates of GDM, preexisting diabetes mellitus, newly diagnosed diabetes mellitus, GH, preeclampsia, HTN, and PCOS in groups 1–2, but lower rates of preeclampsia in groups 4–5 and HTN in group 4. Regarding the adverse outcomes (Supplementary Tables 3–5), the incidence, ORs and adjusted ORs for most adverse pregnancy outcomes and neonatal outcomes were significantly higher in the LGA subgroup compared to the AGA subgroup in groups 1–2. On the other hand, the LGA subgroup (compared to the AGA subgroup) had lower adjusted ORs for primary cesarean section in group 4, jaundice needing phototherapy/exchange transfusion, respiratory distress, and NICU admission in groups 3–4, newborn sepsis in group 4, ROP in groups 2–4, and BPD in group 4.

## 4 Discussion

### 4.1 Key findings

Our study found that the risks associated with LGA were significantly higher for several adverse perinatal outcomes in term and late-preterm neonates (groups 1–2) but not in moderate to extremely preterm neonates (groups 3–5). These adverse outcomes included primary cesarean section, prolonged or obstructed labor, neonatal hypoglycemia, birth trauma, HIE, jaundice, respiratory distress, NICU admission, and newborn sepsis. Conversely, for moderate and very preterm neonates, being LGA was associated with lower risks for certain adverse outcomes, including primary cesarean section (group 4), jaundice needing phototherapy/exchange transfusion (groups 3–4), respiratory distress (groups 3–4), NICU admission (groups 3–4), ROP (groups 2–4), and BPD (group 4). These patterns persisted after adjusting for potential confounders. This study pioneers in assessing LGA-related risks across various prematurity levels, revealing that being LGA does not universally indicate increased risks of adverse outcomes in moderate to extremely preterm neonates. These findings underscore the importance of tailoring management strategies for LGA neonates based on gestational age. For instance, while interventions for LGA neonates at term and late preterm should focus on mitigating risks associated with large fetal size, management of LGA neonates born at moderate to extremely preterm should prioritize addressing their developmental vulnerabilities and optimizing neonatal outcomes.

In the literature, the adverse effects of LGA were established mainly from studies in term neonates. A few studies have investigated the impact of LGA on neonatal outcomes in preterm neonates, focusing primarily on prematurity-related morbidities. Boghossian et al. collected data from 156,587 neonates born at 22–29 weeks of gestation in 852 US centers, finding that LGA neonates had decreased risks of mortality, RDS, PDA, NEC, late-onset sepsis, severe ROP, and chronic lung disease compared to AGA neonates but increased risks of early-onset sepsis and severe IVH (8). Similarly, Ozawa et al. reported that being LGA in extremely preterm neonates did not increase the risks of prematurity-related morbidities compared to AGA neonates (10). Baer et al. supported these findings, indicating a significant decrease in preterm morbidity among LGA neonates born prematurely (<37 weeks) (7). Our findings align with these studies, revealing that being LGA in preterm neonates did not increase the risks of most prematurity-related adverse outcomes. Moreover, we showed that the risks of most LGA-related adverse outcomes, including primary cesarean section, prolonged or obstructed labor, and birth trauma, were not significantly higher in moderate to extremely preterm LGA neonates, compared to AGA neonates. Furthermore, the incidence of primary cesarean section, jaundice, respiratory distress, NICU admission, ROP, and BPD, became significantly lower in moderate and very preterm LGA neonates, compared to AGA neonates. These findings suggest that being LGA in moderate to extremely preterm neonates may have neutral or even protective effects on most perinatal outcomes.

On the other hand, our findings revealed that being LGA was associated with increased risks of fetal death across all groups, with the highest risk observed in term neonates (group 1). Previous studies have reported that a significant association between LGA and increased risks of fetal death (17, 18). Notably, Carter et al. (17) found that the risk of stillbirth increased significantly in LGA neonates, particularly in term fetuses, compared with AGA neonates, which supports the findings of the present study. The main causes of stillbirth in LGA neonates included hydrops, placenta abnormalities, cardiac defects, and maternal illness (19). However, some studies have shown conflicting results, with findings suggesting that being LGA was not significantly associated with increased risks of fetal death compared to AGA neonates (20–22). One study even reported reduced risks of fetal death among LGA term neonates (23). Therefore, the relationship between LGA and the risk of fetal death remains inconclusive. In addition, we found that risks of neonatal death were higher in LGA neonates than in AGA neonates in most groups. In contrast, most studies in the literature showed that being LGA did not increase risks of neonatal mortality (10, 22, 24). The main difference between the present study and previous ones is ethnicity, which may indicate distinct pathophysiological mechanisms of neonatal death in Chinese LGA infants. The relationship between LGA and neonatal death warrants further investigation.

Two pivotal studies have compared long-term outcomes between preterm LGA and AGA infants. One study evaluated neurodevelopment at 18–24 months in infants born before 29 weeks of gestation and found no significant differences between the LGA and AGA groups (9). Another study revealed that LGA infants, both term and preterm, did not have higher risks of speech problems in early childhood, compared to AGA infants (25).

### 4.2 Clinical implications

The adverse outcomes observed in term LGA neonates are mainly attributed to large fetal size. In contrast, preterm neonates, even when classified as LGA, are much smaller, and the risks for fetal-size-related adverse outcomes likely diminish progressively as gestational age decreases. Our study highlighted that in preterm groups 2–5, being LGA had a progressively lesser impact on fetal-size-related adverse outcomes, such as primary cesarean section and birth trauma. Furthermore, the risks of outcomes less directly associated with fetal size—including neonatal hypoglycemia, HIE, jaundice, respiratory distress, NICU admission, and newborn sepsis—were no longer higher and, in some cases, even lower (e.g., jaundice, respiratory distress, and NICU admission) in preterm LGA neonates compared to their preterm AGA counterparts.

This phenomenon may partly be explained by factors such as GDM and preexisting diabetes, which contribute to adverse outcomes in term LGA neonates but would exert less influence in preterm births due to shorter intrauterine exposure to hyperglycemia. Our subgroup analysis for neonates born to mothers with and without DIP further supports this inference, as the risk of adverse outcomes between preterm LGA and AGA neonates was not significantly different. However, the protective roles of LGA on jaundice, respiratory distress and NICU admission in groups 3 and 4 were only observed in pregnant women without DIP. This suggests that the pathophysiology of these outcomes may differ between women with and without DIP, warranting further investigation. Another potential explanation for our findings is that preterm LGA neonates, compared to preterm AGA neonates, tend to have more mature organ systems, such as the lungs and liver. This relative maturity may reduce their susceptibility to jaundice, respiratory distress and the likelihood of requiring NICU admission.

Hypertensive disorders of pregnancy, such as preeclampsia, are the another critical contributor to outcomes like HIE, respiratory distress, NICU admission, and newborn sepsis (26–28). In the present study, we found that a higher incidence of hypertensive disorders of pregnancy in mothers of term and late preterm LGA neonates compared to AGA neonates. Conversely, in very and extremely preterm groups, the incidence of hypertensive disorders of pregnancy was lower in mothers of LGA neonates. This observation could be ascribed to the different mechanisms of preeclampsia at early vs. late stages of pregnancy (29, 30). Late-onset preeclampsia often arises from a mismatch between fetal demands and maternal supply. Since LGA neonates have greater fetal demands, it is more likely that term and late preterm LGA neonates would have mismatch between fetal demands and maternal supply and have a higher risk of late-onset preeclampsia. In contrast, early-onset preeclampsia primarily results from placental dysfunction or malperfusion. Since placental dysfunction or malperfusion would be less prevalent in very and extreme preterm LGA neonates, compared to very and extreme preterm AGA neonates, this could explain the lower risk of hypertensive disorders of pregnancy observed in this study. Overall, the inverse relationship between LGA and preeclampsia in very preterm births may mitigate the risks of preeclampsia-related adverse outcomes in preterm LGA neonates. However, factors beyond diabetes in pregnancy and preeclampsia may also contribute to the observed risk patterns, as these patterns remained similar even after adjusting for these variables.

### 4.3 Research implications

The findings of the present study highlight distinct risk patterns associated with LGA in term pregnancies and pregnancies with different degrees of prematurity. While LGA is a significant outcome measure in clinical researches, our results suggest that management strategies for LGA neonates should be tailored to gestational age rather than applying a uniform approach. Future studies should focus on refining management guidelines to address the specific risks and needs of LGA neonates across different prematurity levels, including recommendations for the timing and mode of delivery.

Additionally, the protective effects observed in preterm LGA neonates for certain outcomes warrant further investigation. Understanding the mechanisms behind these effects, such as potential organ maturity advantages or interactions with maternal conditions, could provide critical insights to improve neonatal care and outcomes. By expanding our knowledge in this area, we can better inform clinical practices and optimize the management of LGA neonates born preterm.

### 4.4 Strengths and limitations

The present study has several notable strengths. First, it enrolled a large cohort of 1,602,638 neonates, enabling a comprehensively comparison of maternal and neonatal outcomes across term and various levels of prematurity. Second, appropriate adjustments for baseline maternal characteristics were made, enhancing the robustness and reliability of the evidence presented. However, the study also has some limitations. First, it did not investigate the relationship between LGA and long-term outcomes, which warrants further investigation. Second, the study did not differentiate between spontaneous and iatrogenic causes of preterm deliveries. Additionally, certain variables that may influence preterm birth and pregnancy outcomes—such as anemia, multiparity, prenatal and postnatal weight changes, and dietary habits during pregnancy—were not available in the National Health Insurance Research database (NHIRD) and could not be included in the analysis. These represent valuable directions for future research. Nevertheless, available demographic data, such as maternal age and social background, were adjusted for in the analysis. Finally, the present study enrolled Asian pregnant women only. As a result, the findings should be validated in other ethnic populations to ensure broader applicability.

## 5 Conclusion

Our study highlights that being LGA is associated with increased risks of perinatal complications in term and late preterm neonates, but not in earlier preterm groups. These findings underscore the importance of developing tailored management strategies for LGA neonates that consider the different degrees of prematurity.

## Data Availability

The original contributions presented in the study are included in the article/Supplementary material, further inquiries can be directed to the corresponding authors.
