# Supplementary material for: Re-evaluating large for gestational age: differential effects on perinatal outcomes in term and premature births
Source: Front Med (Lausanne). 2025 Jan 15;11:1498712. doi: 10.3389/fmed.2024.1498712 (PMC11774698; doi:10.3389/fmed.2024.1498712)
Supplement: Supplementary file 1 [file Data_Sheet_1.docx]

**Supplementary Figure 1. Flowchart depicting enrollment of the participants in the study.** Large for gestational age (LGA) and appropriate for gestational age (AGA) are defined by the Taiwan growth standard. SGA, small for gestational age.

Singleton ≥24 weeks and ≤42 weeks of gestational age at delivery from Birth Certificate Application in 2007-2018, N=2,226,022

Excluded, n=283,442

1. Maternal age <18 or >55 years, n=6,977

2. Neonate with sex unknown, n=11

3. Neonate with SGA, n=209,788

4. Neonate with congenital anomaly, n=66,666

Enrolled, N=1,942,580

LGA, N=143,761

8.3%

AGA, N=1,555,612

91.7%

LGA, N=163,526

AGA, N=1,779,054

Excluded, N=243,207

1. Neonate ID missing or born in 2018, n=241,646

2. Twice delivery records in a year, n=1,561

Enrolled, N=1,699,373

**Supplementary Table 1.** **Incidence of pregnancy outcomes in pregnant women with LGA or AGA neonates, divided into 5 groups according to gestational age (GA) at delivery*.**

|  | Group 1  GA 37-42 weeks | | Group 2  GA 34-36 weeks | | Group 3  GA 32-33 weeks | | Group 4  GA 28-31 weeks | | Group 5  GA 24-27 weeks | |
| --- | --- | --- | --- | --- | --- | --- | --- | --- | --- | --- |
|  | LGA | AGA | LGA | AGA | LGA | AGA | LGA | AGA | LGA | AGA |
| **Common adverse pregnancy outcomes** |  |  |  |  |  |  |  |  |  |  |
| Primary cesarean section ^1,2,4^ | **36.51^§^** | **20.91** | **32.13^§^** | **26.71** | 38.25 | 38.89 | ***39.39^§^*** | ***51.01*** | 39.26 | 46.83 |
| Prolonged or obstructed labor ^1,2^ | **10.33^§^** | **6.97** | **5.16^§^** | **3.03** | 1.20 | 0.67 | NA | 0.29 | 0.00 | NA |
| **Common adverse neonatal outcomes** |  |  |  |  |  |  |  |  |  |  |
| Neonatal hypoglycemia ^1,2^ | **1.41^§^** | **0.16** | **3.41^§^** | **1.69** | 5.38 | 4.21 | 3.67 | 2.35 | NA | 1.24 |
| Birth trauma (shoulder dystocia, brachial plexus injury, clavicular fracture) ^1,2^ | **1.24^§^** | **0.27** | **0.95^§^** | **0.14** | NA | 0.09 | 0.00 | NA | 0.00 | 0.00 |
| Hypoxic ischemic encephalopathy ^1,2^ | **0.17^§^** | **0.11** | **0.51^†^** | **0.30** | 1.39 | 0.90 | 0.61 | 1.16 | 2.22 | 2.04 |
| Jaundice needing phototherapy or exchange transfusion ^1,2,3,4^ | **32.82^§^** | **31.57** | **48.49^§^** | **45.56** | ***55.78^§^*** | ***67.27*** | ***67.55^§^*** | ***80.39*** | 70.37 | 71.27 |
| Respiratory distress ^1,2,3,4^ | **0.67^§^** | **0.30** | **5.50^†^** | **4.83** | ***27.89^§^*** | ***36.99*** | ***50.82^§^*** | ***61.38*** | 62.96 | 69.87 |
| NICU admission ^1,2,3,4^ | **3.00^§^** | **1.64** | **13.84^§^** | **10.28** | ***49.00^§^*** | ***58.07*** | ***76.94^§^*** | ***88.15*** | 87.41 | 89.96 |
| Fetal death‖ ^1,2,3,4,5^ | **0.11^§^** | **0.03** | **0.73^§^** | **0.30** | **3.63^‡^** | **1.82** | **6.70^‡^** | **4.34** | **26.54^§^** | **14.43** |
| Neonatal death (≤ 28 days) ^1,2,3,4^ | **0.06^†^** | **0.04** | **0.30^†^** | **0.18** | **3.19^§^** | **1.01** | **6.94^‡^** | **3.89** | 32.59 | 30.61 |
| **Common adverse outcomes in prematurity** |  |  |  |  |  |  |  |  |  |  |
| Newborn sepsis ^1,2^ | **3.84^§^** | **3.17** | **8.95^‡^** | **7.86** | 19.72 | 20.89 | 24.49 | 28.53 | 39.26 | 36.68 |
| Retinopathy of prematurity ^2,3,4^ | 0.00 | <0.001 | ***0.16^§^*** | ***0.56*** | ***6.37^§^*** | ***12.11*** | ***18.37^§^*** | ***26.52*** | 22.96 | 24.65 |
| Intraventricular hemorrhage | 0.04 | 0.03 | 0.35 | 0.30 | 1.79 | 3.27 | 6.12 | 6.82 | 11.85 | 9.67 |
| Necrotizing enterocolitis | 0.01 | 0.01 | NA | 0.10 | NA | 1.01 | 3.06 | 3.20 | 2.22 | 5.64 |
| Bronchopulmonary dysplasia ^4^ | NA | <0.001 | NA | 0.02 | 0.60 | 0.40 | ***5.71^§^*** | ***9.93*** | 17.04 | 23.63 |

Note: Definitions of LGA and AGA are according to the WHO growth standard. Data were presented as percentage.

AGA, appropriate for gestational age; LGA, large for gestational age; NA, not applicable due to rare events of the outcome in this group; NICU, neonatal intensive care unit; WHO, World Health Organization.

* Group 1: term (gestational age ≥37 and ≤42 weeks); Group 2: late preterm (gestational age ≥34 and <37 weeks); Group 3: moderate preterm (gestational age ≥32 and <34 weeks); Group 4: very preterm (gestational age ≥28 and <32 weeks); Group 5: extremely preterm (≥24 and <28 weeks).

1~5 indicates the number of group with significant difference between LGA and AGA.

† p value <0.05.

‡ p value <0.01.

§ p value <0.001.

‖ Subjects with missing neonatal ID, subjects whose neonates were born in 2018, and subjects with twice delivery records in a year were included for analysis.

**Supplementary Table 2. Baseline characteristics of pregnant women with LGA neonates and AGA neonates, divided into 5 groups according to** **gestational age (GA) at delivery*.**

|  | Group 1  GA 37-42 weeks | |  | Group 2  GA 34-36 weeks | |  | Group 3  GA 32-33 weeks | |  | Group 4  GA 28-31 weeks | |  | Group 5  GA 24-27 weeks | |  |
| --- | --- | --- | --- | --- | --- | --- | --- | --- | --- | --- | --- | --- | --- | --- | --- |
|  | LGA | AGA | p-value | LGA | AGA | p-value | LGA | AGA | p-value | LGA | AGA | p-value | LGA | AGA | p-value |
| N (%) | 132845 (8.37) | 1455058 (91.63) |  | 9678 (10.10) | 86175 (89.90) |  | 644 (7.86) | 7550 (92.14) |  | 450 (8.15) | 5072 (91.85) |  | 144 (7.57) | 1757 (92.43) |  |
|  |  |  |  |  |  |  |  |  |  |  |  |  |  |  |  |
| Maternal age (years) | 31.93  ±4.67 | 30.93  ±4.70 | **<.0001** | 32.37  ±4.97 | 31.25  ±5.07 | **<.0001** | 31.85  ±5.56 | 31.62  ±5.21 | 0.3254 | 31.85  ±5.67 | 31.99  ±5.22 | 0.6346 | 31.99  ±5.51 | 32.05  ±5.17 | 0.8983 |
|  |  |  |  |  |  |  |  |  |  |  |  |  |  |  |  |
| Neonatal sex (%) |  |  |  |  |  |  |  |  |  |  |  |  |  |  |  |
| Male | 52.61 | 51.54 | **<.0001** | 57.76 | 56.45 | **0.0138** | 57.14 | 57.83 | 0.7355 | 62.44 | 56.66 | **0.0176** | 55.56 | 56.00 | 0.9169 |
| Female | 47.39 | 48.46 |  | 42.24 | 43.55 |  | 42.86 | 42.17 |  | 37.56 | 43.34 |  | 44.44 | 44.00 |  |
| GA (weeks) | 38.48  ±1.06 | 38.61  ±1.04 | **<.0001** | 35.63  ±0.63 | 35.57  ±0.67 | **<.0001** | 32.61  ±0.49 | 32.61  ±0.49 | 0.9481 | 29.75  ±1.09 | 29.86  ±1.09 | **0.0291** | 25.85  ±1.11 | 25.72  ±1.12 | 0.1945 |
|  |  |  |  |  |  |  |  |  |  |  |  |  |  |  |  |
| BW (g) | 3823  ±248 | 3134  ±273 | **<.0001** | 3379  ±318 | 2627  ±273 | **<.0001** | 2696  ±299 | 1990  ±228 | **<.0001** | 1992  ±355 | 1470  ±247 | **<.0001** | 1199  ±275 | 865  ±157 | **<.0001** |
|  |  |  |  |  |  |  |  |  |  |  |  |  |  |  |  |
| GDM (%) | 16.77 | 12.15 | **<.0001** | 21.40 | 13.14 | **<.0001** | 20.03 | 13.50 | **<.0001** | 15.33 | 11.30 | **0.0104** | 5.56 | 4.10 | 0.5208 |
| Preexisting diabetes mellitus (%) | 1.54 | 0.44 | **<.0001** | 5.97 | 0.90 | **<.0001** | 7.76 | 1.66 | **<.0001** | 6.44 | 2.03 | **<.0001** | 2.78 | 1.31 | 0.2656 |
| Newly diagnosed diabetes mellitus in pregnancy (%) | 0.46 | 0.14 | **<.0001** | 1.54 | 0.30 | **<.0001** | 2.17 | 0.46 | **<.0001** | 1.78 | 0.45 | **0.0005** | 2.08 | 0.28 | 0.0043 |
| GH (%) | 3.43 | 2.00 | **<.0001** | 9.38 | 4.99 | **<.0001** | 9.63 | 7.84 | 0.1084 | 6.89 | 9.19 | 0.1025 | 4.17 | 5.35 | 0.5408 |
| Preeclampsia (%) | 2.57 | 1.55 | **<.0001** | 7.88 | 4.76 | **<.0001** | 9.01 | 9.36 | 0.7644 | *4.89* | *10.43* | ***0.0002*** | *1.39* | *5.86* | ***0.0239*** |
| HTN (%) | 1.89 | 1.24 | **<.0001** | 5.97 | 3.62 | **<.0001** | 9.63 | 7.02 | **0.0142** | *5.11* | *9.01* | ***0.0049*** | 3.47 | 6.26 | 0.1772 |
| PCOS (%) | 1.59 | 1.24 | **<.0001** | 2.14 | 1.63 | **0.0002** | 2.17 | 1.76 | 0.4492 | 1.56 | 2.05 | 0.4734 | 0.00 | 1.82 | 0.1024 |
| Living area† (%) |  |  |  |  |  |  |  |  |  |  |  |  |  |  |  |
| Urban | 74.92 | 75.48 | **<.0001** | 70.92 | 74.96 | **<.0001** | 76.24 | 82.69 | **0.0002** | 79.11 | 87.09 | **<.0001** | 80.56 | 87.48 | **0.0453** |
| Suburban | 22.05 | 22.05 |  | 25.90 | 22.48 |  | 19.10 | 14.62 |  | 16.89 | 10.15 |  | 13.89 | 8.20 |  |
| Rural | 2.92 | 2.32 |  | 3.09 | 2.45 |  | 4.66 | 2.68 |  | 4.00 | 2.74 |  | 5.56 | 4.33 |  |
| Monthly family income (NTD) (%) |  |  |  |  |  |  |  |  |  |  |  |  |  |  |  |
| ≤20100 | 19.18 | 19.56 | **<.0001** | 23.36 | 21.97 | **<.0001** | 29.04 | 23.38 | **<.0001** | 27.78 | 22.52 | **0.0032** | 31.94 | 20.77 | **0.0102** |
| 20101-22800 | 23.73 | 23.66 |  | 25.52 | 24.18 |  | 21.58 | 23.21 |  | 20.67 | 23.74 |  | 20.83 | 24.02 |  |
| 22801-42000 | 29.63 | 31.68 |  | 26.17 | 29.95 |  | 24.53 | 29.25 |  | 28.67 | 29.14 |  | 25.69 | 30.17 |  |
| >42000 | 20.17 | 20.45 |  | 15.07 | 18.65 |  | 15.84 | 18.69 |  | 14.44 | 18.83 |  | 12.50 | 18.61 |  |

Note: Definitions of LGA and AGA are according to the Taiwan growth standard. Data were presented as mean ± standard deviation or percentage.

AGA, appropriate for gestational age; BW, birth weight; GA, gestational age; GDM, gestational diabetes mellitus; GH, gestational hypertension; HTN, hypertension; LGA, large for gestational age; NTD, New Taiwan Dollar; PCOS, polycystic ovary syndrome.

* Group 1: term (gestational age ≥37 and ≤42 weeks); Group 2: late preterm (gestational age ≥34 and <37 weeks); Group 3: moderate preterm (gestational age ≥32 and <34 weeks); Group 4: very preterm (gestational age ≥28 and <32 weeks); Group 5: extremely preterm (≥24 and <28 weeks).

† Urban (metropolis), suburban (satellite cities), exurban or rural areas.

**Supplementary Table 3. Incidence of pregnancy outcomes in pregnant women with LGA or AGA neonates, divided into 5 groups according to gestational age (GA) at delivery*.**

|  | Group 1  GA 37-42 weeks | | Group 2  GA 34-36 weeks | | Group 3  GA 32-33 weeks | | Group 4  GA 28-31 weeks | | Group 5  GA 24-27 weeks | |
| --- | --- | --- | --- | --- | --- | --- | --- | --- | --- | --- |
|  | LGA | AGA | LGA | AGA | LGA | AGA | LGA | AGA | LGA | AGA |
| **Common adverse pregnancy outcomes** |  |  |  |  |  |  |  |  |  |  |
| Primary cesarean section ^1,2,4^ | **30.63^§^** | **20.14** | **29.98^§^** | **26.41** | 36.96 | 37.36 | ***38.22^§^*** | ***47.85*** | 40.28 | 45.59 |
| Prolonged or obstructed labor ^1,2,3^ | **10.30^§^** | **6.64** | **4.95^§^** | **2.96** | **1.71^‡^** | **0.64** | NA | 0.32 | 0.00 | NA |
| **Common adverse neonatal outcomes** |  |  |  |  |  |  |  |  |  |  |
| Neonatal hypoglycemia ^1,2,4^ | **0.65^§^** | **0.15** | **2.53^§^** | **1.64** | 5.59 | 4.07 | **3.78^†^** | **2.29** | NA | 1.2 |
| Birth trauma (shoulder dystocia, brachial plexus injury, clavicular fracture) ^1,2^ | **0.88^§^** | **0.23** | **0.68^§^** | **0.13** | NA | 0.09 | 0.00 | NA | 0.00 | 0.00 |
| Hypoxic ischemic encephalopathy ^1^ | **0.14^§^** | **0.11** | 0.40 | 0.29 | 1.55 | 0.87 | 0.67 | 1.18 | 2.08 | 2.11 |
| Jaundice needing phototherapy or exchange transfusion ^1,3,4^ | **32.27^§^** | **31.45** | 46.36 | 45.63 | ***57.14^§^*** | ***66.98*** | ***66.00^§^*** | ***80.07*** | 70.14 | 71.26 |
| Respiratory distress ^1,3,4^ | **0.47^§^** | **0.29** | 4.43 | 4.78 | ***27.17^§^*** | ***36.87*** | ***48.89^§^*** | ***61.46*** | 65.28 | 69.61 |
| NICU admission ^1,2,3,4^ | **2.27^§^** | **1.63** | **10.94^†^** | **10.18** | ***48.60^§^*** | ***57.47*** | ***75.56^§^*** | ***87.68*** | 88.89 | 89.76 |
| Fetal death‖ ^1,2,3,4,5^ | **0.06^§^** | **0.03** | **0.46^‡^** | **0.29** | **2.95^†^** | **1.71** | **7.29^§^** | **4.11** | **24.89^§^** | **14.33** |
| Neonatal death (≤ 28 days)^1,2,3,4^ | **0.05^‡^** | **0.04** | **0.26^†^** | **0.17** | **2.95^§^** | **0.93** | **7.33^§^** | **3.81** | 32.64 | 30.45 |
| **Common adverse outcomes in prematurity** |  |  |  |  |  |  |  |  |  |  |
| Newborn sepsis ^1,4^ | **3.56^§^** | **3.16** | 7.83 | 7.84 | 20.81 | 20.85 | ***24.00^†^*** | ***28.71*** | 40.28 | 36.54 |
| Retinopathy of prematurity ^2,3,4^ | <.0.001 | <0.001 | ***0.17^§^*** | ***0.54*** | ***6.06^§^*** | ***11.88*** | ***18.00^§^*** | ***26.04*** | 20.83 | 24.93 |
| Intraventricular hemorrhage ^3^ | 0.04 | 0.03 | 0.33 | 0.28 | ***1.71^†^*** | ***3.19*** | 5.56 | 6.72 | 12.50 | 9.68 |
| Necrotizing enterocolitis | 0.01 | 0.01 | 0.06 | 0.10 | NA | 0.99 | 3.11 | 3.15 | 2.78 | 5.63 |
| Bronchopulmonary dysplasia ^4^ | <0.001 | <0.001 | NA | 0.03 | 0.47 | 0.40 | ***5.33^‡^*** | ***9.37*** | 15.28 | 23.51 |

Note: Definitions of LGA and AGA are according to the Taiwan growth standard. Data were presented as percentage.

AGA, appropriate for gestational age; LGA, large for gestational age; NA, not applicable due to rare events of the outcome in this group; NICU, neonatal intensive care unit.

* Group 1: term (gestational age ≥37 and ≤42 weeks); Group 2: late preterm (gestational age ≥34 and <37 weeks); Group 3: moderate preterm (gestational age ≥32 and <34 weeks); Group 4: very preterm (gestational age ≥28 and <32 weeks); Group 5: extremely preterm (≥24 and <28 weeks).

1~5 indicates the number of group with significant difference between LGA and AGA.

† p value <0.05.

‡ p value <0.01.

§ p value <0.001.

‖ Subjects with missing neonatal ID, subjects whose neonates were born in 2018, and subjects with twice delivery records in a year were included for analysis.

**Supplementary Table 4. Crude odds ratios (95% confidence intervals) for pregnancy outcomes of pregnant women with LGA neonates, compared to pregnant women with AGA neonates, divided into 5 groups according to gestational age (GA) at delivery*.**

|  | Group 1  GA 37-42 weeks | Group 2  GA 34-36 weeks | Group 3  GA 32-33 weeks | Group 4  GA 28-31 weeks | Group 5  GA 24-27 weeks |
| --- | --- | --- | --- | --- | --- |
| **Common adverse pregnancy outcomes** |  |  |  |  |  |
| Primary cesarean section | **1.75 (1.73-1.77)^§^** | **1.19 (1.14-1.25)^§^** | 0.98 (0.83-1.16) | ***0.67 (0.55-0.82)^§^*** | 0.81 (0.57-1.14) |
| Prolonged or obstructed labor | **1.61 (1.58-1.65)^§^** | **1.70 (1.54-1.88)^§^** | **2.72 (1.40-5.26)^‡^** | 1.41 (0.32-6.16) | **1.61 (1.58-1.65)^§^** |
| **Common adverse neonatal outcomes** |  |  |  |  |  |
| Neonatal hypoglycemia | **4.36 (4.03-4.72)^§^** | **1.56 (1.36-1.78)^§^** | 1.40 (0.98-1.99) | 1.68 (0.99-2.82) | 1.16 (0.27-5.02) |
| Birth trauma (shoulder dystocia, brachial plexus injury, clavicular fracture) | **3.87 (3.62-4.14)^§^** | **5.42 (3.99-7.37)^§^** | 1.68 (0.21-13.64) | NA | NA |
| Hypoxic ischemic encephalopathy | **1.31 (1.13-1.52)^§^** | 1.37 (0.98-1.93) | 1.79 (0.92-3.50) | 0.56 (0.18-1.80) | 0.99 (0.30-3.25) |
| Jaundice needing phototherapy or exchange transfusion | **1.04 (1.03-1.05)^§^** | 1.03 (0.99-1.07) | ***0.66 (0.56-0.77)^§^*** | ***0.48 (0.39-0.59)^§^*** | 0.95 (0.65-1.37) |
| Respiratory distress | **1.64 (1.51-1.79)^§^** | 0.92 (0.84-1.02) | ***0.64 (0.53-0.77)^§^*** | ***0.60 (0.49-0.73)^§^*** | 0.82 (0.57-1.17) |
| NICU admission | **1.40 (1.35-1.46)^§^** | **1.08 (1.01-1.16)^†^** | ***0.70 (0.60-0.82)^§^*** | ***0.43(0.35-0.55)^§^*** | 0.91 (0.53-1.57) |
| Fetal death‖ | **2.01 (1.60-2.53)^§^** | **1.63 (1.21-2.19)^‡^** | **1.75 (1.11-2.75)^†^** | **1.83 (1.30-2.59)^§^** | **1.98 (1.43-2.74)^§^** |
| Neonatal death (≤ 28 days) | **1.42 (1.10-1.84)^§^** | **1.54 (1.01-2.35)^†^** | **3.25 (1.94-5.43)^§^** | **2.00 (1.37-2.93)^§^** | 1.11 (0.77-1.59) |
| **Common adverse outcomes in prematurity** |  |  |  |  |  |
| Newborn sepsis | **1.13 (1.10-1.17)^§^** | 1.00 (0.92-1.08) | 1.00 (0.82-1.22) | ***0.78 (0.63-0.98)^†^*** | 1.17 (0.83-1.66) |
| Retinopathy of prematurity | 1.62 (0.57-4.64) | ***0.30 (0.18-0.50)^§^*** | ***0.48 (0.34-0.67)^§^*** | ***0.62 (0.49-0.80)^§^*** | 0.79 (0.52-1.20) |
| Intraventricular hemorrhage | 1.31 (0.98-1.74) | 1.16 (0.81-1.68) | ***0.53 (0.29-0.97)^†^*** | 0.82 (0.54-1.24) | 1.33 (0.79-2.24) |
| Necrotizing enterocolitis | 0.90 (0.50-1.62) | 0.64 (0.28-1.47) | 0.31 (0.08-1.27) | 0.99 (0.57-1.72) | 0.48 (0.17-1.32) |
| Bronchopulmonary dysplasia | 1.24 (0.53-2.89) | 0.41 (0.06-3.00) | 1.17 (0.36-3.86) | ***0.55 (0.36-0.83)^‡^*** | ***0.59 (0.37-0.94)^†^*** |

Note: Definitions of LGA and AGA are according to the Taiwan growth standard.

AGA, appropriate for gestational age; LGA, large for gestational age; NA, not applicable due to rare events of the outcome in this group; NICU, neonatal intensive care unit.

* Group 1: term (gestational age ≥37 and ≤42 weeks); Group 2: late preterm (gestational age ≥34 and <37 weeks); Group 3: moderate preterm (gestational age ≥32 and <34 weeks); Group 4: very preterm (gestational age ≥28 and <32 weeks); Group 5: extremely preterm (≥24 and <28 weeks).

† p value <0.05.

‡ p value <0.01.

§ p value <0.001.

‖ Subjects with missing neonatal ID, subjects whose neonates were born in 2018, and subjects with twice delivery records in a year were included for analysis.

**Supplementary Table 5. Adjusted odds ratios (95% confidence intervals) for pregnancy outcomes of pregnant women with LGA neonates, compared to pregnant women with AGA neonates, divided into 5 groups according to gestational age (GA) at delivery*.**

|  | Group 1  GA 37-42 weeks | Group 2  GA 34-36 weeks | Group 3  GA 32-33 weeks | Group 4  GA 28-31 weeks | Group 5  GA 24-27 weeks |
| --- | --- | --- | --- | --- | --- |
| **Common adverse pregnancy outcomes** |  |  |  |  |  |
| Primary cesarean section | **1.73 (1.71-1.75)^§^** | **1.10 (1.05-1.15)^§^** | 0.97 (0.81-1.15) | ***0.70 (0.57-0.86)^§^*** | 0.84 (0.59-1.21) |
| Prolonged or obstructed labor | **1.68 (1.65-1.71)^§^** | **1.75 (1.59-1.93)^§^** | **2.58 (1.36-4.89)^‡^** | 1.98 (0.57-6.89) | NA |
| **Common adverse neonatal outcomes** |  |  |  |  |  |
| Neonatal hypoglycemia | **3.78 (3.49-4.10)^§^** | **1.37 (1.19-1.57)^§^** | 1.42 (0.99-2.03) | **1.70 (1.01-2.88)^†^** | 1.18 (0.27-5.19) |
| Birth trauma (shoulder dystocia, brachial plexus injury, clavicular fracture) | **3.70 (3.46-3.96)^§^** | **5.18 (3.78-7.11)^§^** | 1.32 (0.16-11.12) | NA | NA |
| Hypoxic ischemic encephalopathy | **1.26 (1.08-1.47)^‡^** | 1.26 (0.89-1.78) | **2.07 (1.05-4.08)^†^** | 0.55 (0.17-1.78) | 0.86 (0.25-2.88) |
| Jaundice needing phototherapy or exchange transfusion | **1.02 (1.01-1.04)^§^** | 0.99 (0.95-1.04) | ***0.64 (0.54-0.76)^§^*** | ***0.49 (0.39-0.60)^§^*** | 1.02 (0.70-1.49) |
| Respiratory distress | **1.52 (1.40-1.66)^§^** | ***0.83 (0.75-0.92)^§^*** | ***0.62 (0.52-0.75)^§^*** | ***0.60 (0.49-0.73)^§^*** | 0.86 (0.60-1.23) |
| NICU admission | **1.34 (1.29-1.39)^§^** | 1.00 (0.93-1.07) | ***0.68 (0.58-0.80)^§^*** | ***0.45 (0.35-0.57)^§^*** | 1.01 (0.58-1.75) |
| Fetal death‖ | **1.77 (1.40-2.24)^§^** | **1.52 (1.12-2.07)^‡^** | **1.62 (1.60-1.63)^§^** | **1.77 (1.24-2.51)^‡^** | **1.86 (1.32-2.62)^§^** |
| Neonatal death (≤ 28 days) | **1.36 (1.05-1.76)^†^** | 1.45 (0.94-2.24) | **3.55 (2.10-5.98)^§^** | **2.00 (1.35-2.95)^§^** | 1.05 (0.72-1.52) |
| **Common adverse outcomes in prematurity** |  |  |  |  |  |
| Newborn sepsis | **1.12 (1.09-1.16)^§^** | 0.95 (0.88-1.03) | 0.96 (0.79-1.18) | ***0.73 (0.58-0.91)^‡^*** | 1.16 (0.82-1.64) |
| Retinopathy of prematurity | 1.60 (0.56-4.60) | ***0.31 (0.19-0.51)^§^*** | ***0.48 (0.35-0.68)^§^*** | ***0.63 (0.49-0.81)^§^*** | 0.79 (0.52-1.21) |
| Intraventricular hemorrhage | 1.23 (0.93-1.64) | 1.06 (0.73-1.54) | 0.55 (0.30-1.02) | 0.78 (0.51-1.19) | 1.36 (0.80-2.29) |
| Necrotizing enterocolitis | 0.89 (0.50-1.61) | 0.63 (0.27-1.46) | 0.28 (0.07-1.16) | 0.91 (0.52-1.59) | 0.45 (0.16-1.25) |
| Bronchopulmonary dysplasia | 1.19 (0.51-2.77) | 0.45 (0.06-3.35) | 1.28 (0.39-4.23) | ***0.58 (0.38-0.89)^†^*** | 0.64 (0.40-1.03) |

Note: Definitions of LGA and AGA are according to the Taiwan growth standard.

AGA, appropriate for gestational age; LGA, large for gestational age; NA, not applicable due to rare events of the outcome in this group; NICU, neonatal intensive care unit.

Models were adjusted for maternal age, gestational age, neonatal sex, diabetes in pregnancy (defined as gestational diabetes mellitus, preexisting diabetes mellitus, or newly diagnosed diabetes mellitus), hypertensive disorders of pregnancy (defined as gestational hypertension, preeclampsia, or chronic hypertension), polycystic ovary syndrome, living area, and monthly family income.

* Group 1: term (gestational age ≥37 and ≤42 weeks); Group 2: late preterm (gestational age ≥34 and <37 weeks); Group 3: moderate preterm (gestational age ≥32 and <34 weeks); Group 4: very preterm (gestational age ≥28 and <32 weeks); Group 5: extremely preterm (≥24 and <28 weeks).

† p value <0.05.

‡ p value <0.01.

§ p value <0.001.

‖ Subjects with missing neonatal ID, subjects whose neonates were born in 2018, and subjects with twice delivery records in a year were included for analysis.

**Supplementary Table 6. Adjusted** **odds ratios (95% confidence intervals) for pregnancy outcomes of pregnant women with LGA neonates and diabetes in pregnancy, compared to pregnant women with AGA neonates and diabetes in pregnancy, divided into 5 groups according to gestational age (GA) at delivery*. Definitions of LGA and AGA are according to the WHO growth standard.**

|  | Group 1  GA 37-42 weeks | Group 2  GA 34-36 weeks | Group 3  GA 32-33 weeks | Group 4  GA 28-31 weeks | Group 5  GA 24-27 weeks |
| --- | --- | --- | --- | --- | --- |
| **Common adverse pregnancy outcomes** |  |  |  |  |  |
| Primary cesarean section | **2.15 (2.05-2.24)^§^** | **1.20 (1.08-1.33)^§^** | 1.02 (0.72-1.45) | 0.71 (0.47-1.08) | 3.57 (0.94-13.52) |
| Prolonged or obstructed labor | **1.37 (1.27-1.47)^§^** | **1.49 (1.16-1.90)^‡^** | 0.99 (0.12-8.02) | 13.63 (0.46-405.09) | NA |
| **Common adverse neonatal outcomes** |  |  |  |  |  |
| Neonatal hypoglycemia | **6.72 (5.76-7.84)^§^** | **2.17 (1.71-2.76)^§^** | 1.68 (0.82-3.45) | 1.15 (0.33-4.04) | NA |
| Birth trauma (shoulder dystocia, brachial plexus injury, clavicular fracture) | **3.95 (3.32-4.71)^§^** | **6.41 (3.50-11.72)^§^** | 11.25 (0.49-257.04) | NA | NA |
| Hypoxic ischemic encephalopathy | **1.63 (1.03-2.59)*^†^*** | 1.28 (0.66-2.48) | NA | 0.74 (0.09-6.20) | NA |
| Jaundice needing phototherapy or exchange transfusion | **1.22 (1.17-1.27)^§^** | **1.33 (1.20-1.47)^§^** | 0.92 (0.64-1.32) | 0.77 (0.49-1.23) | 1.72 (0.41-7.25) |
| Respiratory distress | **3.30 (2.70-4.03)^§^** | **1.22 (1.02-1.47)^†^** | 0.82 (0.58-1.17) | 0.96 (0.64-1.44) | 1.99 (0.48-8.28) |
| NICU admission | **2.55 (2.31-2.83)^§^** | **1.57 (1.37-1.79)^§^** | 0.83 (0.59-1.17) | 0.95 (0.54-1.67) | NA |
| Fetal death‖ | **4.90 (3.03-7.94)^§^** | **2.29 (1.25-4.19)^‡^** | 1.69 (0.57-5.04) | 1.74 (0.72-4.22) | 0.78 (0.12-5.13) |
| Neonatal death (≤ 28 days) | **2.91 (1.56-5.42)^§^** | 1.93 (0.82-4.55) | 2.04 (0.54-7.68) | **2.17 (1.05-4.47)^†^** | 0.49 (0.09-2.60) |
| **Common adverse outcomes in prematurity** |  |  |  |  |  |
| Newborn sepsis | **1.34 (1.22-1.47)^§^** | **1.18 (1.01-1.39)^†^** | 0.93 (0.62-1.41) | 0.69 (0.44-1.10) | 1.22 (0.35-4.22) |
| Retinopathy of prematurity | NA | 0.43 (0.16-1.18) | 0.66 (0.38-1.17) | 0.66 (0.40-1.08) | 2.74 (0.84-8.96) |
| Intraventricular hemorrhage | **2.46 (1.21-4.98)^†^** | 0.59 (0.23-1.49) | 0.20 (0.03-1.49) | 1.14 (0.54-2.40) | 1.14 (0.19-6.82) |
| Necrotizing enterocolitis | 2.70 (0.60-12.08) | 0.55 (0.07-4.29) | 0.42 (0.05-3.20) | 0.72 (0.25-2.09) | 0.90 (0.08-10.49) |
| Bronchopulmonary dysplasia | NA | NA | 1.93 (0.17-22.04) | 0.47 (0.18-1.22) | 1.32 (0.33-5.26) |

Note: Definitions of LGA and AGA are according to the WHO growth standard.

Abbreviation: AGA, appropriate for gestational age; DIP, diabetes in pregnancy; LGA, large for gestational age; NA, not applicable due to rare events of the outcome in this group; NICU, neonatal intensive care unit; WHO, World Health Organization.

Models were adjusted for maternal age, gestational age, neonatal sex, hypertensive disorders of pregnancy (defined as gestational hypertension, preeclampsia, or chronic hypertension), polycystic ovary syndrome, living area, and monthly family income.

* Group 1: term (gestational age ≥37 and ≤42 weeks); Group 2: late preterm (gestational age ≥34 and <37 weeks); Group 3: moderate preterm (gestational age ≥32 and <34 weeks); Group 4: very preterm (gestational age ≥28 and <32 weeks); Group 5: extremely preterm (≥24 and <28 weeks).

† p value <0.05.

‡ p value <0.01.

§ p value <0.001.

‖ Subjects with missing neonatal ID, subjects whose neonates were born in 2018, and subjects with twice delivery records in a year were included for analysis.

**Supplementary Table 7. Adjusted odds ratios (95% confidence intervals) for pregnancy outcomes of pregnant women with LGA neonates and without diabetes in pregnancy, compared to pregnant women with AGA neonates and without diabetes in pregnancy, divided into 5 groups according to gestational age (GA) at delivery*. Definitions of LGA and AGA are according to the WHO growth standard.**

|  | Group 1  GA 37-42 weeks | Group 2  GA 34-36 weeks | Group 3  GA 32-33 weeks | Group 4  GA 28-31 weeks | Group 5  GA 24-27 weeks |
| --- | --- | --- | --- | --- | --- |
| **Common adverse pregnancy outcomes** |  |  |  |  |  |
| Primary cesarean section | **2.12 (2.07-2.17)^§^** | **1.13 (1.04-1.22)^‡^** | 0.98 (0.78-1.24) | ***0.68 (0.54-0.85)^§^*** | ***0.67 (0.45-0.99)^†^*** |
| Prolonged or obstructed labor | **1.64 (1.57-1.70)^§^** | **1.93 (1.65-2.27)^§^** | 2.24 (0.88-5.70) | 0.74 (0.10-5.70) | NA |
| **Common adverse neonatal outcomes** |  |  |  |  |  |
| Neonatal hypoglycemia | **7.50 (6.63-8.49)^§^** | **1.39 (1.09-1.78)^‡^** | 1.19 (0.72-1.97) | 1.68 (0.96-2.93) | 1.50 (0.34-6.64) |
| Birth trauma (shoulder dystocia, brachial plexus injury, clavicular fracture) | **4.44 (3.95-5.00)^§^** | **5.98 (3.84-9.29)^§^** | NA | NA | NA |
| Hypoxic ischemic encephalopathy | **1.52 (1.14-2.03)^‡^** | 1.51 (0.88-2.60) | 2.06 (0.93-4.53) | 0.46 (0.11-1.89) | 1.22 (0.36-4.11) |
| Jaundice needing phototherapy or exchange transfusion | 0.98 (0.95-1.00) | 0.95 (0.88-1.02) | ***0.51 (0.41-0.64)^§^*** | ***0.46 (0.37-0.58)^§^*** | 0.99 (0.66-1.48) |
| Respiratory distress | **1.55 (1.30-1.84)^§^** | 0.85 (0.71-1.02) | ***0.58 (0.45-0.75)^§^*** | ***0.58 (0.47-0.72)^§^*** | 0.71 (0.48-1.04) |
| NICU admission | **1.44 (1.34-1.56)^§^** | 1.07 (0.96-1.20) | ***0.62 (0.50-0.78)^§^*** | ***0.40 (0.31-0.51)^§^*** | 0.76 (0.44-1.31) |
| Fetal death‖ | **2.38 (1.59-3.58)^§^** | **2.29 (1.52-3.44)^§^** | **2.33 (1.39-3.91)^‡^** | **1.47 (1.01-2.16)^†^** | **2.00 (1.41-2.85)^§^** |
| Neonatal death (≤ 28 days) | 1.04 (0.57-1.89) | 1.36 (0.66-2.78) | **3.81 (2.07-6.99)^§^** | **1.72 (1.11-2.76)^†^** | 1.12 (0.75-1.66) |
| **Common adverse outcomes in prematurity** |  |  |  |  |  |
| Newborn sepsis | **1.13 (1.06-1.21)^§^** | 1.01 (0.88-1.16) | 0.87 (0.66-1.15) | ***0.77 (0.60-0.98)^†^*** | 1.07 (0.73-1.57) |
| Retinopathy of prematurity | NA | ***0.23 (0.09-0.62)^‡^*** | ***0.41 (0.25-0.67)^§^*** | ***0.62 (0.47-0.82)^§^*** | 0.77 (0.48-1.24) |
| Intraventricular hemorrhage | 0.87 (0.43-1.76) | 1.30 (0.72-2.33) | 0.73 (0.36-1.48) | 1.22 (0.69-2.19) | 1.26 (0.70-2.26) |
| Necrotizing enterocolitis | 0.67 (0.17-2.72) | 0.31 (0.04-2.23) | 0.29 (0.04-2.13) | 0.96 (0.51-1.80) | 0.28 (0.07-1.14) |
| Bronchopulmonary dysplasia | 1.96 (0.47-8.10) | NA | 1.41 (0.33-5.95) | ***0.62 (0.40-0.95)^†^*** | 0.68 (0.41-1.13) |

Note: Definitions of LGA and AGA are according to the WHO growth standard.

Abbreviation: AGA, appropriate for gestational age; DIP, diabetes in pregnancy; LGA, large for gestational age; NA, not applicable due to rare events of the outcome in this group; NICU, neonatal intensive care unit; WHO, World Health Organization.

Models were adjusted for maternal age, gestational age, neonatal sex, hypertensive disorders of pregnancy (defined as gestational hypertension, preeclampsia, or chronic hypertension), polycystic ovary syndrome, living area, and monthly family income.

* Group 1: term (gestational age ≥37 and ≤42 weeks); Group 2: late preterm (gestational age ≥34 and <37 weeks); Group 3: moderate preterm (gestational age ≥32 and <34 weeks); Group 4: very preterm (gestational age ≥28 and <32 weeks); Group 5: extremely preterm (≥24 and <28 weeks).

† p value <0.05.

‡ p value <0.01.

§ p value <0.001.

‖ Subjects with missing neonatal ID, subjects whose neonates were born in 2018, and subjects with twice delivery records in a year were included for analysis.

**Supplementary Table 8. Adjusted odds ratios (95% confidence intervals) for pregnancy outcomes of pregnant women with LGA neonates and diabetes in pregnancy, compared to pregnant women with AGA neonates and diabetes in pregnancy, divided into 5 groups according to gestational age (GA) at delivery*. Definitions of LGA and AGA are according to the Taiwan growth standard.**

|  | Group 1  GA 37-42 weeks | Group 2  GA 34-36 weeks | Group 3  GA 32-33 weeks | Group 4  GA 28-31 weeks | Group 5  GA 24-27 weeks |
| --- | --- | --- | --- | --- | --- |
| **Common adverse pregnancy outcomes** |  |  |  |  |  |
| Primary cesarean section | **1.84 (1.79-1.89)^§^** | **1.14 (1.04-1.25)^†^** | 1.09 (0.79-1.51) | 0.68 (0.44-1.04) | 3.26 (0.87-12.15) |
| Prolonged or obstructed labor | **1.54 (1.48-1.62)^§^** | **1.51 (1.22-1.86)^§^** | 3.03 (0.75-12.23) | 14.08 (0.50-395.95) | NA |
| **Common adverse neonatal outcomes** |  |  |  |  |  |
| Neonatal hypoglycemia | **4.25 (3.71-4.87)^§^** | **1.85 (1.48-2.30)^§^** | 1.83 (0.95-3.53) | 1.38 (0.39-4.96) | NA |
| Birth trauma (shoulder dystocia, brachial plexus injury, clavicular fracture) | **3.44 (3.00-3.94)^§^** | **4.72 (2.58-8.64)^§^** | 10.61 (0.46-244.05) | NA | NA |
| Hypoxic ischemic encephalopathy | 1.33 (0.96-1.84) | 1.40 (0.78-2.52) | NA | 0.81 (0.10-6.63) | NA |
| Jaundice needing phototherapy or exchange transfusion | **1.13 (1.10-1.16)^§^** | **1.25 (1.15-1.37)^§^** | 0.87 (0.62-1.22) | 0.80 (0.50-1.29) | 1.58 (0.37-6.72) |
| Respiratory distress | **2.27 (1.94-2.66)^§^** | 1.03 (0.87-1.21) | ***0.72 (0.52-0.99)^†^*** | 0.90 (0.59-1.36) | 1.98 (0.47-8.27) |
| NICU admission | **1.82 (1.68-1.96)^§^** | **1.29 (1.15-1.46)^§^** | 0.79 (0.57-1.08) | 0.96 (0.54-1.71) | NA |
| Fetal death‖ | **2.35 (1.51-3.66)^§^** | 1.47 (0.82-2.66) | 1.41 (0.47-4.22) | 1.86 (0.76-4.53) | 0.82 (0.13-5.22) |
| Neonatal death (≤ 28 days) | **1.82 (1.09-3.04)^†^** | 1.89 (0.87-4.11) | 3.36 (0.94-12.03) | **2.39 (1.15-4.98)^†^** | 0.57 (0.11-3.07) |
| **Common adverse outcomes in prematurity** |  |  |  |  |  |
| Newborn sepsis | **1.20 (1.13-1.28)^§^** | 1.06 (0.92-1.21) | 0.82 (0.55-1.21) | 0.69 (0.43-1.11) | 1.16 (0.33-4.03) |
| Retinopathy of prematurity | 1.71 (0.19-15.44) | ***0.41 (0.18-0.95)^†^*** | 0.65 (0.38-1.11) | 0.78 (0.48-1.29) | 2.66 (0.81-8.73) |
| Intraventricular hemorrhage | 1.07 (0.56-2.03) | 0.84 (0.42-1.68) | 0.17 (0.02-1.22) | 0.88 (0.38-2.02) | 1.19 (0.20-7.19) |
| Necrotizing enterocolitis | 1.39 (0.40-4.85) | 0.33 (0.04-2.55) | 0.37 (0.05-2.88) | 0.78 (0.27-2.27) | 0.63 (0.05-8.20) |
| Bronchopulmonary dysplasia | NA | NA | 1.33 (0.12-15.48) | 0.55 (0.21-1.43) | 1.29 (0.33-5.08) |

Note: Definitions of LGA and AGA are according to the Taiwan growth standard.

AGA, appropriate for gestational age; DIP, diabetes in pregnancy; LGA, large for gestational age; NA, not applicable due to rare events of the outcome in this group; NICU, neonatal intensive care unit.

Models were adjusted for maternal age, gestational age, neonatal sex, hypertensive disorders of pregnancy (defined as gestational hypertension, preeclampsia, or chronic hypertension), polycystic ovary syndrome, living area, and monthly family income.

* Group 1: term (gestational age ≥37 and ≤42 weeks); Group 2: late preterm (gestational age ≥34 and <37 weeks); Group 3: moderate preterm (gestational age ≥32 and <34 weeks); Group 4: very preterm (gestational age ≥28 and <32 weeks); Group 5: extremely preterm (≥24 and <28 weeks).

† p value <0.05.

‡ p value <0.01.

§ p value <0.001.

‖ Subjects with missing neonatal ID, subjects whose neonates were born in 2018, and subjects with twice delivery records in a year were included for analysis.

**Supplementary Table 9. Adjusted odds ratios (95% confidence intervals) for pregnancy outcomes of pregnant women with LGA neonates and without diabetes in pregnancy, compared to pregnant women with AGA neonates and without diabetes in pregnancy, divided into 5 groups according to gestational age (GA) at delivery*. Definitions of LGA and AGA are according to the Taiwan growth standard.**

|  | Group 1  GA 37-42 weeks | Group 2  GA 34-36 weeks | Group 3  GA 32-33 weeks | Group 4  GA 28-31 weeks | Group 5  GA 24-27 weeks |
| --- | --- | --- | --- | --- | --- |
| **Common adverse pregnancy outcomes** |  |  |  |  |  |
| Primary cesarean section | **1.70 (1.68-1.72)^§^** | **1.08 (1.03-1.15)^‡^** | 0.94 (0.76-1.15) | ***0.70 (0.56-0.89)^‡^*** | 0.76 (0.52-1.11) |
| Prolonged or obstructed labor | **1.66 (1.63-1.70)^§^** | **1.83 (1.63-2.05)^§^** | **2.83 (1.31-6.11)^‡^** | 0.77 (0.10-5.93) | NA |
| **Common adverse neonatal outcomes** |  |  |  |  |  |
| Neonatal hypoglycemia | **3.51 (3.17-3.88)^§^** | 1.09 (0.90-1.32) | 1.26 (0.81-1.95) | 1.76 (0.99-3.14) | 1.44 (0.32-6.40) |
| Birth trauma (shoulder dystocia, brachial plexus injury, clavicular fracture) | **3.78 (3.49-4.09)^§^** | **5.20 (3.58-7.54)^§^** | NA | NA | NA |
| Hypoxic ischemic encephalopathy | **1.24 (1.05-1.48)^†^** | 1.16 (0.75-1.79) | **2.33 (1.18-4.58)^†^** | 0.49 (0.12-2.03) | 1.02 (0.30-3.47) |
| Jaundice needing phototherapy or exchange transfusion | 1.00 (0.99-1.01) | ***0.91 (0.87-0.96)^§^*** | ***0.57 (0.47-0.69)^§^*** | ***0.42 (0.33-0.53)^§^*** | 0.99 (0.67-1.47) |
| Respiratory distress | **1.32 (1.19-1.46)^§^** | ***0.73 (0.64-0.84)^§^*** | ***0.58 (0.46-0.72)^§^*** | ***0.53 (0.43-0.66)^§^*** | 0.80 (0.55-1.17) |
| NICU admission | **1.21 (1.16-1.27)^§^** | ***0.87 (0.80-0.95)^‡^*** | ***0.64 (0.53-0.78)^§^*** | ***0.37 (0.29-0.48)^§^*** | 0.92 (0.52-1.61) |
| Fetal death‖ | **1.58 (1.19-2.09)^‡^** | **1.53 (1.07-2.19)^†^** | **1.99 (1.20-3.30)^‡^** | **1.74 (1.19-2.56)^‡^** | **1.94 (1.37-2.75)^§^** |
| Neonatal death (≤ 28 days) | 1.23 (0.91-1.66) | 1.24 (0.73-2.13) | **3.58 (2.01-6.36)^§^** | **1.87 (1.17-2.99)^‡^** | 1.11 (0.76-1.64) |
| **Common adverse outcomes in prematurity** |  |  |  |  |  |
| Newborn sepsis | **1.10 (1.06-1.14)^§^** | ***0.90 (0.82-0.99)^†^*** | 1.03 (0.81-1.30) | ***0.74 (0.57-0.96)^†^*** | 1.14 (0.79-1.65) |
| Retinopathy of prematurity | 1.57 (0.47-5.26) | ***0.27 (0.14-0.51)^§^*** | ***0.40 (0.26-0.62)^§^*** | ***0.59 (0.44-0.79)^§^*** | 0.65 (0.40-1.05) |
| Intraventricular hemorrhage | 1.27 (0.92-1.74) | 1.15 (0.74-1.80) | 0.72 (0.38-1.36) | 0.75 (0.46-1.23) | 1.32 (0.76-2.29) |
| Necrotizing enterocolitis | 0.79 (0.40-1.56) | 0.74 (0.30-1.85) | 0.24 (0.03-1.71) | 0.98 (0.51-1.88) | 0.39 (0.12-1.26) |
| Bronchopulmonary dysplasia | 1.61 (0.68-3.79) | NA | 1.08 (0.25-4.58) | ***0.57 (0.36-0.92)^†^*** | ***0.57 (0.34-0.96)^†^*** |

Note: Definitions of LGA and AGA are according to the Taiwan growth standard.

AGA, appropriate for gestational age; DIP, diabetes in pregnancy; LGA, large for gestational age; NA, not applicable due to rare events of the outcome in this group; NICU, neonatal intensive care unit.

Models were adjusted for maternal age, gestational age, neonatal sex, hypertensive disorders of pregnancy (defined as gestational hypertension, preeclampsia, or chronic hypertension), polycystic ovary syndrome, living area, and monthly family income.

* Group 1: term (gestational age ≥37 and ≤42 weeks); Group 2: late preterm (gestational age ≥34 and <37 weeks); Group 3: moderate preterm (gestational age ≥32 and <34 weeks); Group 4: very preterm (gestational age ≥28 and <32 weeks); Group 5: extremely preterm (≥24 and <28 weeks).

† p value <0.05.

‡ p value <0.01.

§ p value <0.001.

‖ Subjects with missing neonatal ID, subjects whose neonates were born in 2018, and subjects with twice delivery records in a year were included for analysis.
